# Effect of two major health reforms on health care cost and utilization in Fars Province of Iran: family physician program and health transformation plan

**DOI:** 10.1186/s12913-020-05257-8

**Published:** 2020-05-06

**Authors:** Mohsen Bayati, Khosro Keshavarz, Farhad Lotfi, Abbas KebriaeeZadeh, Omid Barati, Shahram Zareian, Akbar Amiri, Sajad Delavari

**Affiliations:** 1 Health Human Resources Research Center, School of Management and Information Sciences, Shiraz University of Medical Sciences, Shiraz, Iran; 2 Department of Pharmacoeconomics and Pharmaceutical Administration, School of Pharmacy, Pharmaceutical Management & Economics Research Center, Tehran University of Medical Sciences, Tehran, Iran; 3 Hospital Management Research Center, Education Development Center, Iran University of Medical Sciences, Tehran, Iran; 4 Social Security Organization, Shiraz, Iran

**Keywords:** Health care reform, Utilization, Health care costs, Family physicians, Health policy, Interrupted time series analysis, Universal coverage

## Abstract

**Background:**

Family physician program (FPP) and health transformation plan (HTP) are two major reforms that have been implemented in Iran’s health system in recent. The present study was aimed at evaluating the impact of these two reforms on the level of service utilization and cost of health care services.

**Methods:**

This longitudinal study was conducted on people insured by social security organization in Fars province during 2009–2016. The data on the utilization of services and costs of general practitioner visits, specialist visits, medications, imaging, laboratory tests, and hospitalization were collected. Interrupted time series analysis was used to analyze the effect of the two mentioned reforms.

**Results:**

FPP resulted in a significant reduction in the number of specialist visits, imaging, and laboratory tests in the short term, and in the number of radiology services, laboratory tests, and hospitalization in the long term. In contrast, HTP significantly increased the utilization of radiology services and laboratory tests both in the short term and long term. Concerning the costs, FPP resulted in a reduction in costs in short and long term except general practitioners’ and specialist visit, and medication in long term. However, HTP resulted in an increase in health care costs in both of the studied time periods.

**Conclusions:**

FPP has been successful in rationalizing the utilization of services. On the other hand, HTP has improved people’s access to services by increasing the utilization; but it has increased health care costs. Therefore, policymakers must adopt an agenda to revise and re-design the plan.

## Background

For several years, universal health coverage by 2030 has been the most important slogan of health systems [1, 2]. Universal health coverage means that all people can receive the health services they need without suffering financial problems [3]. The countries across the world have different status in terms of the universal health coverage. Countries that are pioneer in this field have made various reforms in the domains of financing and health services delivery to achieve universal health coverage. In Iran, various reforms have been implemented to increase people’s access to quality services, reduce out-of-pocket payments, and increase population coverage. In the past decade, two important reforms have been launched in the health system of Iran, including family physician program (FPP) and health transformation plan (HTP).

**FPP:** FPP was launched in rural areas in 2005 through providing insurance coverage for all people living in villages and towns under 20,000 populations and through allocating about 6000 general practitioners (GPs). In this program, GPs were introduced as the focal point in the referral system and the access of rural people to them was increased [4]. Seven years after the implementation of FPP in rural areas, FPP in urban areas, as a pilot plan, was started in Fars and Mazandaran provinces in 2012 [5]. In this program, GPs are recruited as family physicians; they supervise a health team consisting of midwives, nurses, nutritionists, etc. People can choose their own family physician. Each physician covers about 2000 to 3000 people and they are paid by capitation mechanism. Family physicians are responsible for providing first-level services (primary care) and, if necessary, referring patients to higher levels [5, 6].

**HTP:** Despite the advancements in the delivery of primary care, the provision of the second and third levels of services had faced numerous challenges [7]. The high level of out-of-pocket payout (more than 50%) and the inappropriate quality of and inadequate access to hospital and specialized services were among the main motives for designing HTP in 2014 [8]. The plan was implemented in several phases and through several policy packages. The main objectives of the plan were to reduce out-of-pocket payments for inpatient services through increasing government’s and insurance companies’ share in the costs of services provided for patients referring to public hospitals, increase people’s access to public-health services through implementing policy packages to increase the physicians’ residence time, and improve the quality of hoteling and visits in public hospitals [8]. Furthermore, a plan to provide free public insurance coverage for individuals without an insurance scheme was implemented in order to increase the financial protection for the people [8]. Due to the lack of funding and lack of reform in the plan to respond changes and insufficiencies, it faced some problems after the early years.

One of the major steps in the health system reform is the assessment of reforms, making efforts to revise the program to address deficiencies, and making progress towards the designated goals. Changes in the utilization of services and their costs are among the main implications of policies and programs in the health sector that need to be assessed. The aim of this study was to investigate the impact of these two major reforms in the health system over the past decade in Iran. In other words, this study evaluated the impact of implementing FPP in urban areas and HTP on the utilization of different health care services as well as their costs.

## Methods

The present study was conducted using the data on people insured by Social Security Insurance Organization in Fars province. Fars Province, with a population of 4.8 million people, is the fourth most populated province of Iran. Social Security Organization is the largest health insurance provider in Iran, and more than half of the country’s population is covered by its insurance scheme. Given the fact that urban FPP was implemented only in Fars province and Mazandaran province, and because of data availability in Fars province, the study was conducted on population insured by social security organization in Fars province. According to the latest information, more than two million and a hundred thousand people are covered by this insurer in Fars province.

Using census method, the data on the utilization and cost of health services were collected for the entire insured people. The studied subjects (total sample size) in 2009, 2010, 2011, 2012, 2013, 2014, 2015, and 2016 were 1,596,813, 1,677,289, 1,800,210, 1,974,275, 2,029,048, 2,026,650, 2,109,731, and 2,105,295, respectively. The studied data were classified into two major categories: the data on the utilization of health services and on the cost of health services. The variables of utilization and cost of services were collected and classified by general practitioner visit, specialist visits, medication, imaging, laboratory tests, and hospitalization. The variables of utilization of general practitioner visits and the cost of hospitalization were excluded from the analysis due to the lack or shortage of data at some points of time. Data were collected monthly from the first month of 2009 to the last month of 2016.

Interrupted time series (ITS) analysis was used to investigate the effect of implementing FPP and HTP on the utilization and costs of health care services. This analysis method is an appropriate technique for analyzing the effect of corrective policies in the health sector and has been used by various researchers. Using this method, the impact of the policy is measurable in the short term as well as in the long term. Thus, segmented regression was used to conduct interrupted time series analysis. In a standard ITS model, the segmented regression is as follows:

$$ {Y}_t={\beta}_0+{\beta}_1\;T+{\beta}_2\;{X}_t+{\beta}_3\;{TX}_t $$

Where Y_t_ is the outcome, T_t_ is the time trend that shows the number of months, X_t_ is a dummy variable that is equal to 0 for the years before the intervention and equal to 1 for the years after the intervention, TX_t_ is the interaction between T_t_ and X_t_ that shows the time trend after the intervention. The model coefficients b0-b3, respectively, represent the primary level of outcome, the trend of outcome before the intervention, the changes in the level of outcome after the intervention, and the changes in the trend after the intervention. In other words, b2 represent the short-term effect and b3 shows the long-term effects of the intervention in the outcome. Finally, e_t_ represent the disturbance term in the model.

In our study, the effect of two reforms in the health sector on the utilization and cost of various health care services was investigated. To achieve the mentioned goal, the following model was developed:

$$ Health\ services\ utilization/{costs}_{\mathrm{t}}={\beta}_0+{\beta}_1\ {\mathrm{Trend}}_{\mathrm{t}}+{\beta}_2{\mathrm{FPP}}_{\mathrm{t}}+{\beta}_3\mathrm{Post}\ \mathrm{FPP}\ {\mathrm{Trend}}_{\mathrm{t}}+{\beta}_4{\mathrm{HTP}}_{\mathrm{t}}+{\beta}_5\mathrm{Post}\ \mathrm{HTP}\ {\mathrm{Trend}}_{\mathrm{t}}+{\nu}_{\mathrm{t}} $$

Where *Health services utilization*/*costs*_t_, as the outcome, is a number of variables related to the utilization and cost of services that were previously mentioned; the abovementioned model was estimated for each of them.

To control inflation effect on health services costs variables, we adjusted it according to the consumer price index which is reported by Central Bank of Iran [9].

As shown in the model, β_0_ and β_1_ show the initial level of service cost / service utilization, and the trend of service cost / utilization. In addition, β_2_ to β_5_, respectively, represent the short-term effect of FPP, the long-term effect of FPP, the short-term effect of HTP, and the long-term effect of HTP. It should be mentioned that β_2_ and β_4_ coefficients which are changes in intercept after FPP and HTP interventions respectively, show the short term effect. Moreover, β_3_ and β_5_ coefficients which are changes in the slope of regression line after FTP and HTP interventions respectively, indicate the long term effect. The short term effect is the immediate change, measured 1 month after intervention which is July 2012 for FPP and June 2014 for HTP. The long-term effect for FPP is the effect from first month after intervention to May 2014 which HTP starts and for HTP is the effect from the first month after intervention to the end of the study which is March 2017.

Durbin-Watson test was used for checking auto-correlation and the results of test does not reveal any concern on auto-correlation. Augmented Dickey Fuller (ADF) test and Engle and Granger co-integration test was performed to check the stationary of the variables and co-integration of the models to avoid spurious regression.

## Results

According to the ADF stationarity test (Table 1) all utilization variables were stationary, so there was not any concern about spurious regression in utilization models. However, cost variables were non-stationary, so we applied Engel and Granger co-integration approach for cost variables equations. Fortunately, according to the Engel and Granger co-integration approach, all cost models’ residuals were stationary (bottom of the Table 3). So there was also no concern about spurious regression in cost models.

**Table 1:** Augmented Dickey Fuller stationarity test results for the utilization and cost variables

	With intercept	Significance	With intercept and trend	Significance
SPvisit	−3.269	0.0163	−5.501	0.0000
Medication	−5.178	0.0000	−5.959	0.0000
Radiology	−3.070	0.0289	−3.627	0.0277
Laboratory	−4.100	0.0010	−4.589	0.0011
Inpatient Admission	−3.841	0.0025	−8.081	0.0000
GPvisitC	−0.845	0.8056	−2.137	0.5253
SPvisitC	−1.062	0.7299	−2.932	0.1522
MedicationC	−0.936	0.7758	−2.984	0.1366
RadiologyC	−1.369	0.5971	−2.252	0.4607
LaboratoryC	−1.787	0.3868	−2.845	0.1810

The effect of the two reforms on the utilization of services (Table 2) and then their effects on the cost of health services (Table 3) are presented.

**Table 2:** The effect of two major health system reforms on health care utilization in Iran

		SP visit	Medication	Radiology	Laboratory	Inpatient Admission
Pre-intervention	Initial level	151,144.4 (0.000)	587,891.2 (0.000)	29,321.6 (0.000)	110,572.1 (0.000)	13,100.79 (0.000)
	Initial trend	992.35 (0.000)	619.71 (0.507)	1778.59 (0.000)	790.84 (0.000)	132.11 (0.000)
Post-FPP	Trend after FPP	− 598.00 (0.586)	3233.41 (0.065)	− 828.19 (0.035)	− 881.69 (0.206)	16.91 (0.728)
	Change in level after FPP (short-term)	− 47,011.85 (0.002)	−19,344.09 (0.545)	−23,328.64 (0.000)	−19,224.63 (0.042)	755.01 (0.239)
	Change in trend after FPP (long-term)	− 1590.35 (0.159)	2613.7 (0.188)	− 2606.79 (0.000)	− 1672.53 (0.021)	−115.20 (0.028)
Post-HTP	Trend after HTP	−50.35 (0.756)	3693.98 (0.001)	598.68 (0.000)	1004.21 (0.000)	53.70 (0.006)
	Change in level after HTP (short-term)	−20,034.09 (0.174)	−50,537.56 (0.135)	20,702.44 (0.003)	29,598.61 (0.005)	391.73 (0.658)
	Change in trend after HTP (long-term)	547.65 (0.622)	460.57 (0.824)	1426.88 (0.001)	1885.90 (0.001)	36.78 (0.483)
Model significance	F-statistics	95.65 (0.000)	6.72 (0.000)	61.41 (0.000)	23.15 (0.000)	84.37 (0.000)
	Adjusted R^2^	0.831	0.222	0.758	0.533	0.812

*SP* Specialist

*HTP* Health Transformation Plan

*FPP* Family Physician Program

**Table 3:** The effect of two major health system reforms on cost of health sevices in Iran

		GPvisitC	SPvisitC	MedicationC	RadiologyC	LaboratoryC
Pre-intervention	Initial level	92.97 (0.000)	104.66 (0.000)	749.37 (0.000)	50.76 (0.000)	147.89 (0.000)
	Initial trend	1.01 (0.000)	1.23 (0.000)	−1.36 (0.194)	3.65 (0.000)	1.92 (0.000)
Post-FPP	Trend after FPP	32.33 (0.000)	4.97 (0.000)	31.85 (0.000)	−2.54 (0.000)	−1.57 (0.147)
	Change in level after FPP (short-term)	70.23 (0.127)	−52.37 (0.000)	− 187.41 (0.000)	−52.24 (0.000)	−42.14 (0.015)
	Change in trend after FPP (long-term)	31.32 (0.000)	3.73 (0.001)	33.22 (0.000)	−6.19 (0.000)	−3.49 (0.003)
Post-HTP	Trend after HTP	7.18 (0.000)	3.86 (0.000)	16.85 (0.000)	6.51 (0.000)	6.19 (0.000)
	Change in level after HTP (short-term)	− 149.30 (0.002)	72.64 (0.000)	−5.32 (0.939)	75.16 (0.000)	101.85 (0.000)
	Change in trend after HTP (long-term)	−25.15 (0.000)	−1.10 (0.333)	−15 (0.000)	9.06 (0.000)	7.77 (0.000)
Model significance	F-statistics	726.18 (0.000)	359.52 (0.000)	357.18 (0.000)	141.88 (0.000)	105.34 (0.000)
	Adjusted R2	0.974	0.949	0.949	0.881	0.846
Engel and Granger co-integration*	With intercept	−3.797 (0.003)	−7.365 (0.000)	−7.048 (0.000)	−4.253 (0.000)	−6.237 (0.000)
	With intercept and trend	−3.768 (0.018)	− 7.322 (0.000)	− 7.013 (0.000)	− 4.217 (0.004)	−6.187 (0.000)

*GP* General Practitioner

*SP* Specialist

*C* Cost

*HTP* Health Transformation Plan

*FPP* Family Physician Program

* ADF stationarity test on residuals

FPP mainly reduced the utilization of health services in the short term. Accordingly, the number of specialist visits, imaging, and laboratory tests utilized by people were significantly decreased immediately after the initiation of FPP. Moreover, in the short term, there was an insignificant decrease in the number of prescribed drugs and an insignificant increase in the number of hospitalization. In the long term, FPP has resulted in a reduction in the utilization of most services, and the number of radiology services, laboratory tests, and hospitalization decreased significantly. In addition, there was an insignificant reduction in specialist visits, and an insignificant increase in prescribed drugs.

HTP resulted in a significant increase in the use of radiology services and laboratory test. However, in the long term, this plan increased the utilization of all health services. HTP, in the long term, significantly increased the utilization of radiology and laboratory services and insignificantly increased the hospitalization, medications, and specialist visits. The results of F test showed that all utilization models were significant at a significance level of 1%.

**Fig. 1:**
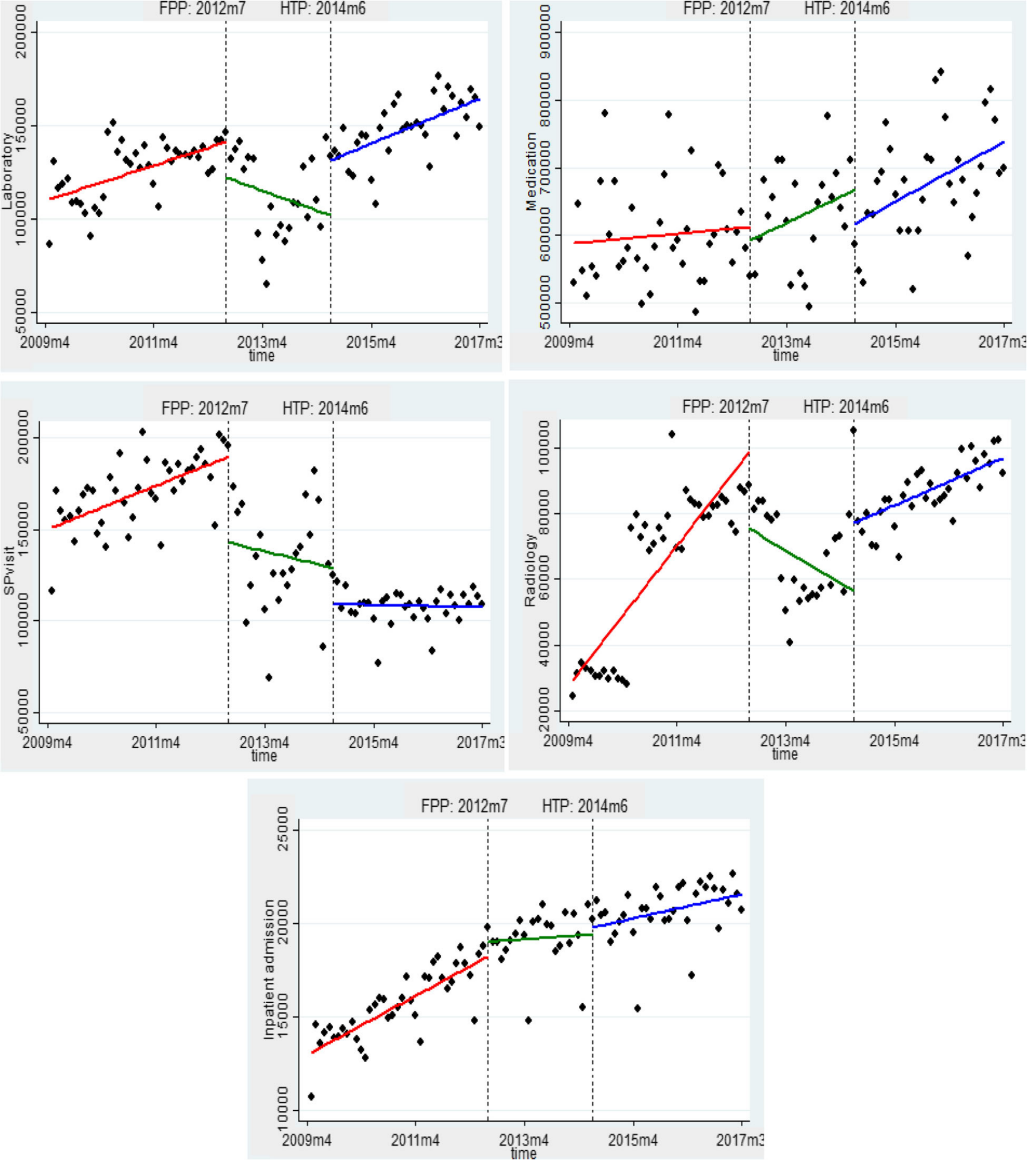
The effect FPP and HTP on the utilization of various health services

The effect of these two reforms on the utilization of various health services is presented in Fig. 1.

FPP mainly reduced costs in the short term. After the launch of this program, there was a significant decrease in the costs of specialist visits, drugs, radiology services, and laboratory tests. In the long term, the effects of the plan were almost the opposite of its short-term effects. Accordingly, in the long term, the cost of general practitioner visits, specialist visits, and medications increased significantly. Moreover, the cost of radiology services and laboratory tests decreased significantly.

**Fig. 2:**
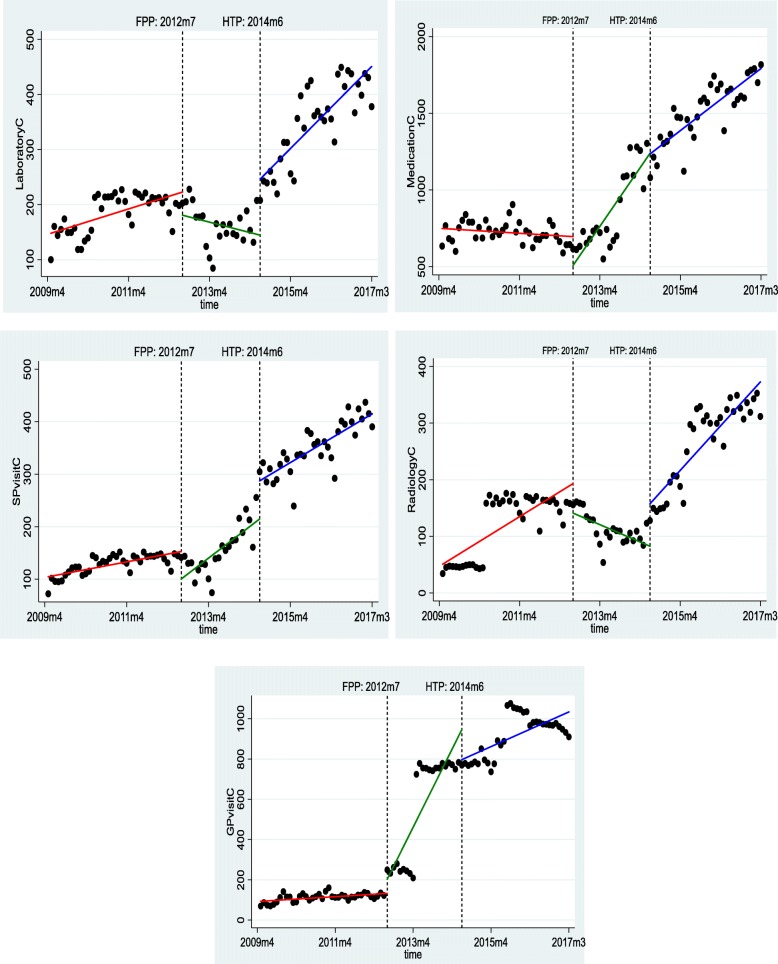
The effect FPP and HTP on the utilization of various health costs

HTP mainly increased the costs in the short term. After the launch of this plan, there was a significant increase in the cost of specialist visit, radiology services, and laboratory tests. However, the cost of general practitioner visits decreased significantly. In the long term, HTP significantly increased the costs of radiology services and laboratory tests. In addition, there was a significant reduction in the costs of general practitioner visits and medications. The results of F test showed that all cost models were significant at a significance level of 1% (Table 3 and Fig. 2).

## Discussion

Based on the findings of this study, FPP has led to a significant reduction in the utilization of health services both in the short term and long term. The findings showed that the number of specialist visits, number of imaging services, and number of laboratory tests utilized by population covered by Social Security Insurance were significantly reduced immediately after the implementation of this program. This decline is also evident in long-term horizons, and FPP has been able to significantly reduce the utilization of imaging services, laboratory tests, and hospitalizations. Although there has been an increase in the utilization of some services in the short term or long term, these increases have not been significant.

FPP, which was aimed at better management of the referral system, has reduced the number of referrals to specialized levels. According to the findings of other studies, it can be observed that FPP can help improve quality of health care services and reduce their cost. For example, Simo et al. (2010) found that referrals to family physicians not only improved the prescription practices, but also reduced the financial burden to the patient and the community [10]. In addition, according to the results of a study in Iran, the implementation of FPP led to the relative progress in the delivery of family health services including women’s periodic examinations and pap smear sampling, and increased referral to the physician for medical services, that indicated improvements in the quality of services [11].

It is worth noting that FPP has not been able to reduce hospitalization in the short term, rather it has experienced an insignificant relative increase. In the short term, the access to family physicians might have helped to identify many of the unmet medical needs of the people that have resulted in referral to higher levels. However, in the long term, there has been a significant decrease in hospitalization. This finding is not surprising because FPP changes physicians from a therapist to an active health care provider that, along with him, health care providers also observe the health status of the community. Improving people’s access to primary health care and prevention services, in the long term, can reduce the burden of diseases and reduce the need for hospitalization [12, 13]. Improving people’s access to services via the implementation of FPP is a findings that have been cited by many researchers [14–16]. The launch of this project in Iran has helped to increase people’s access to primary health care, especially in rural areas [17]. A review study on 19 papers similarly reported that family physicians can guarantee the durable delivery of services, improved counseling time, improved physician-patient relationship, implementing preventive measures, and ultimately reduce the burden of diseases [18].

On the contrary, HTP has increased the utilization of health services in the short term. The HTP has also significantly increased the utilization of imaging services and laboratory tests both in the short term and the long term; nevertheless, the increase in the utilization of other services including hospitalization services, medicine, and specialist visits has been insignificant. Several studies have confirmed that HTP has increased the utilization of services [19, 20]. Pirozi et al. (2019), for example, confirmed the increased utilization of hospitalization services as a result of the implementation of HTP [21]. Nonetheless, some studies have reported the existence of socioeconomic differences in the utilization of health services after the launch of HTP [22].

In fact, the goal of HTP is somewhat different from that of FPP and, consequently, it has had different achievements. The plan, as one of the major reforms in Iran health system in recent years, has been aimed at removing financial barriers facing service recipients. Therefore, the increase in the utilization of health services and the decrease in out-of-pocket payments [23] are not far from our expectations [24]. This has intensified in the absence of clinical guidelines [25].

Concerning the costs, FPP has led to a reduction in costs in the short term, and the reduction has been significant in the costs of specialist visit, drugs, and radiology services, and laboratory tests. However, in the long term, the effects of the FPP have shown different findings for services. In the long term, the costs of general practitioners visit, specialist visit, and drugs have increased significantly. The cost of radiology services and laboratory tests has also experienced a significant decline both in the short term and the long term.

The reduction in health care expenditure in the short term can be attributed to the rationalization and reduction of service utilization due to the presence of family physicians as health system gatekeepers. In fact, family physicians have made great efforts in controlling referrals, especially at the beginning of the plan, which has reduced the utilization of services at the next levels of the health system. In a comprehensive study, it became clear that FPP could reduce costs and improve the cost-effectiveness of health interventions [18]. However, the increase in costs in the long term which was reported in another study [26] may be attributed to rising inflation in the country in the late 2000s and early 2010s, which has also resulted in an inflation in the health sector [27].

However, in the short term, HTP has mainly led to an increase in costs, except for the cost of a general practitioner visits and drugs. A similar increasing trend in costs in the long term was also observed, but the cost of the general practitioner visits and drugs decreased significantly. The increase in costs, especially the increase in the cost of outpatient services, has also been reported in other studies [28]. In fact, the rise of costs can be justified by the increase in the utilization of services after the launch of HTP [19–21]. On the other hand, this plan increased the share of health system in the general budget and raised health services tariffs based on publishing the book of relative value (which puts new values on health services tariffs), which resulted in a remarkable increase in health system expenditures [19, 29]. Thus, the increase in health service costs can be attributed to the increase in the share of health from GDP and the increase in health care expenditures.

Despite the improvements in people’s access to health services, HTP has failed to be successful in providing financial protection for health services. For example, Homaie Rad et al. (2017) showed that as a result of HTP, out-of-pocket payments for outpatient services increased and the costs of other services did not change significantly. However, there was no significant improvement in equity and utilization indices [28]. In a national study, the amount of exposure to catastrophic health care costs has been reported to be increased from 2.57% in 2008 (before HTP) to 3.25% in 2015 (after HTP) [30]. The same findings were reported in another study [31]. One of the main reasons for this finding is the high emphasis of HTP on inpatient services and the abandonment of outpatient services and services provided in private hospitals [32], while the share of these services in the entire health care system has grown in recent years [33].

This study was carried out on population covered by social security insurance organization, which covers more than half of Iran’s population (about 45 million people). People under the coverage of the Iran health insurance (with a population almost equal to people under the coverage of social security organization) are expected to follow a similar pattern due to relatively similar policies. However, it should be kept in mind that generalizing the research findings to the entire Iranian population (some of which are still not covered by any insurance scheme) should be done with caution.

## Conclusions

The present study is one of the few studies that examine the effects of two important policy interventions in the health system. This study showed that the implementation of FPP in the short term and long term has generally reduced the utilization of health services. Moreover, this decline in service utilization in the short term has led to a reduction in health care costs. However, in long-term we found different findings for various services. On the other hand, HTP, both in the short term and long term, has led to a general increase in utilization and costs of health services.

Hence, reforms and a serious review of HTP should be put on the agenda of policy makers in the health system. In order to improve HTP it is recommended to redesign the payment system, improve the financing system, emphasize on family physician and referral system role in resource allocation and financial flow, and make it necessary to use clinical guidelines to improve the utilization of services and control health care costs.

## Data Availability

The datasets used and/or analyzed during the current study are available from the corresponding author on reasonable request.

## Abbreviations

FPP: Family Physician Program
HTP: Health Transformation Plan
GP: General Practitioner
ITS: Interrupted Time Series
ADF: Augmented Dickey Fuller
SP: Specialist
C: Cost
M: Month
GDP: Gross Domestic Product
